# Executive functions in adults: scoping review of computerized neuropsychological batteries

**DOI:** 10.1590/1980-5764-DN-2025-0374

**Published:** 2026-03-06

**Authors:** Victor Linking Magalhães Campos, Antonio de Pádua Serafim

**Affiliations:** 1 Universidade de São Paulo, Instituto de Psicologia, São Paulo SP, Brazil.

**Keywords:** Neuropsychological Tests, Telediagnostics, Cognition, Executive Function, Testes Neuropsicológicos, Telediagnóstico, Cognição, Função Executiva

## Abstract

Computerized assessment of executive functions (EF) has the potential to increase access to more cost-effective diagnostics, considering the relevance of these skills for quality of life and adaptive functioning.

**Objective:**

This study conducted a scoping review to identify computerized fixed batteries for assessing EF in adults.

**Methods:**

A systematic search was conducted in the United States National Library of Medicine (PubMed), Scopus, Web of Science, Cochrane Library (Reviews), PsychNet, and Scientific Electronic Library Online (SciELO) databases, following the Preferred Reporting Items for Systematic reviews and Meta-Analyses Extension for Scoping Reviews (PRISMA-ScR).

**Results:**

A total of 15,704 records were identified, of which 62 studies met the eligibility criteria. In these, 42 batteries were mapped, which together encompassed 174 tasks associated with executive domains. No battery exclusively assesses EF, and most were developed for computerized application in a supervised face-to-face format. It was also observed that there was no explicit theoretical basis in the construction of the instruments, evidencing important conceptual gaps.

**Conclusion:**

There are fixed computerized batteries applicable to the assessment of EF in adults, although not specific, reflecting the advance of digitalization in neuropsychological practice. The findings highlight the need for cross-cultural adaptations and psychometric validation studies that strengthen the theoretical basis and diagnostic accuracy of these instruments.

## RESUMO.

A informatização da avaliação das funções executivas (FE) tem potencial para ampliar o acesso a diagnósticos com melhor relação custo-benefício, considerando a relevância dessas habilidades para a qualidade de vida e o funcionamento adaptativo.

**Objetivo:**

Este estudo realizou uma revisão de escopo com o objetivo de identificar baterias fixas informatizadas voltadas à avaliação das FE em adultos.

**Métodos:**

A busca sistemática foi conduzida nas bases United States National Library of Medicine (PubMed), Scopus, Web of Science, Cochrane Library (Reviews), PsychNet, and Scientific Electronic Library Online (SciELO), seguindo as diretrizes do *Preferred Reporting Items for Systematic reviews and Meta-Analyses Extension for Scoping Reviews* (PRISMA-ScR).

**Resultados:**

Foram identificados 15.704 registros, dos quais 62 estudos estudos atenderam aos critérios de elegebilidade. Nestes, foram mapeadas 42 baterias, que, em conjunto, englobaram 174 tarefas associadas a domínios executivos. Nenhuma bateria avalia exclusivamente FE, e a maioria foi desenvolvida para aplicação informatizada em formato supervisionado presencial. Observou-se, ainda, ausência de fundamentação teórica explícita na construção dos instrumentos, salientando lacunas conceituais importantes.

**Conclusão:**

Existem baterias fixas informatizadas aplicáveis à avaliação de FE em adultos, ainda que não específicas, refletindo o avanço da digitalização na prática neuropsicológica. Os achados ressaltam a necessidade de adaptações transculturais e estudos de validação psicométrica que fortaleçam a base teórica e a precisão diagnóstica desses instrumentos.

## INTRODUCTION

 Executive functions (EF) encompass a broad range of brain processes that, despite conceptual disagreements in the literature, are widely understood as contributing to intentional and controlled cognitive functioning and goal-directed behavior^1^. There is also consensus that the prefrontal cortex plays a predominant role (relative to other brain regions) in the emergence of these functions^2^. The implications of EF extend across various domains, including Education (mediating teaching-learning processes), Clinical Psychology (as a foundation for mental disorders), and Neuropsychology (with deficits associated with diverse neurological syndromes), highlighting their importance across different populations and contexts^3^. 

 The assessment and diagnosis of EF have increasingly become the focus of research due to their verified associations with mental and physical health conditions, as well as sociodemographic factors^4,5^. Identifying and intervening in EF deficits may slow the progression of related pathologies, underscoring the importance of thorough cognitive evaluations^6^. However, assessment may be hindered by the diversity of definitions attributed to the construct, often resulting in the measurement of distinct cognitive functions based on varied epistemological frameworks, which can limit understanding of executive functioning^7^. 

 The multiplicity of models and theories explaining EF creates a fertile conceptual field but also generates methodological tensions in assessment^4^. Some models stress inhibitory control^1^, others propose relatively independent components^4,5^, while integrative approaches suggest a general supervisory system^3^. This heterogeneity means neuropsychological instruments, though valid within their paradigms, may not align or provide a coherent picture of executive capacities when based on different frameworks. The lack of consensus can lead to diagnostic fragmentation or redundant measures, weakening interpretative foundations^4^. 

 Therefore, it may be helpful to consider explicitly which models guide the instruments chosen in an assessment battery. Tasks such as the Stroop, Tower of London (ToL), or Wisconsin Card Sorting Test embody paradigms that shaped later tests, many of which, despite the similar formats, reflect distinct conceptions of EF^4-6^, such as seeing ToL as an assessment of either planning, cognitive flexibility or Supervisory Attentional System (SAS)^1,7^. A consistent neuropsychological evaluation must balance theoretical coherence with methodological diversity, selecting tools that capture the multidimensional complexity of EF while preserving a clear epistemological basis for solid, reproducible, and scientifically comparable interpretations^7^. Structured tests that cover different EF domains (componential models)^8^ tend to allow for more accurate diagnoses and support personalized intervention strategies based on the specific subsystems of interest^9^. The increasing reliance on cognitive test batteries to assess EF is mainly driven by the recognition that isolated tests often fail to capture the full spectrum of these complex cognitive processes^10^. Comprehensive tools designed to screen various EF not only enhance diagnostic accuracy but also assist in monitoring cognitive changes over time, providing valuable information to clinicians in the management of cognitive deficits^6^. 

 As a result, innovative technologies, such as computerized cognitive batteries aimed at assessing multiple EF domains, have been increasingly incorporated into both research and clinical practice^11^. Among the main advantages of computerized assessment are: increased measurement precision, with reduced errors from manual calculations; the ability to obtain complex variables such as reaction time, which are difficult to measure in traditional settings; greater participant engagement due to familiarity with digital tools; and optimization of professionals’ time, thanks to automated scoring and recording processes, which streamline subsequent stages of evaluation^12^. 

 In the Brazilian context, some computerized instruments for assessing EF are already available, such as tasks based on the Matching Figures paradigm^13^ and the ToL task^14^. However, these instruments typically involve multiple cognitive processes simultaneously, limiting their evaluative specificity. Furthermore, a search conducted in March 2024 in the Psychological Testing Assessment System (*Sistema de Avaliação de Testes Psicológicos* — SATEPSI) of the Federal Council of Psychology (*Conselho Federal de Psicologia* — CFP) found no approved computerized instruments for use with Brazilian adults that are based on componential models of EF, models that are widely cited in the international literature^5^. 

 Given this context, the present study aims to investigate the existence of fixed computerized batteries, available internationally, for the assessment of EF in adults. The study is based on the hypothesis that such batteries are indeed available internationally, considering the growing body of evidence on the advantages of computerized assessment in neuropsychological contexts, particularly in terms of precision, efficiency, and comprehensiveness of cognitive measurement. 

## METHODS

 To achieve this objective, a scoping review of the current literature on fixed computerized batteries designed to assess EF in adults was conducted. The search, selection, and data extraction procedures followed the guidelines of the Preferred Reporting Items for Systematic reviews and Meta-Analyses Extension for Scoping Reviews (PRISMA-ScR)^15^. 

### Information sources and search strategies

 In February 2025, a search was conducted in the databases United States National Library of Medicine (PubMed), Scopus, Web of Science, Cochrane Library (Reviews), PsycNET, and Scientific Electronic Library Online (SciELO). To ensure maximum comprehensiveness, no language restrictions were applied. A time frame starting from 2015 was established to include recent studies that are potentially grounded in updated theoretical models of EF. In all databases — except SciELO — the following descriptors were used: (battery OR questionnaire OR test* OR assessment* OR evaluation OR task* OR instrument* OR inventory* OR screening* OR diagnosis*) AND ("executive functions*" OR executive* OR dysexecutive*) AND (computerized* OR "computerized adaptive testing*" OR "adaptive testing*" OR digital OR remote OR online). In SciELO, equivalent descriptors in Portuguese were applied. The search was conducted in the title, abstract, and keyword fields. Additionally, reverse citation tracking was used by examining the reference lists of included studies to identify other potentially eligible works. Information about the fixed batteries mentioned in the selected studies was gathered by consulting the original articles related to the development of each instrument or those reporting the first available psychometric evidence. In cases where the cited reference did not correspond to the initial publication of the instrument, internal citations within the articles were reviewed to identify and, whenever possible, use the oldest accessible version for the systematic description of each battery. 

### Eligibility criteria

 The inclusion criteria were:

- empirical studies;
- adult participants (aged ≥ 18 years);
- use of fixed batteries aimed at assessing EF (even if also assessing other cognitive functions);
- computerized administration of the battery;
- use of terminology such as "executive function(s)," "executive control," "control," "working memory," "updating," "cognitive flexibility," "shifting," "inhibitory control," "inhibition," "reasoning," "planning," or "problem solving" to describe at least one construct within the battery; and
- scientific articles (i.e., journal papers), based on the rationale that other materials — such as public presentations — may not fully describe all instruments used due to space limitations, and books are often not peer-reviewed.

Exclusion criteria included:

- use of virtual reality;
- no quantitative analysis;
- fixed batteries in which one or more tests or tasks were not administered or were substituted; d) non-article texts (e.g., book chapters, brief reports, editorials);
- paper-and-pencil tests administered digitally without psychometric adaptation;
- unavailable full text;
- non-fixed batteries (i.e., arbitrary test selection by researchers rather than standardized or fixed test sets); and
- studies with participants aged ≤ 18 years.

### Data extraction and analysis

 From the included studies, data were extracted and described based on the SPIDER acronym^16^:

- Sample: study participants;
- Phenomenon of interest: EF and the operational definitions adopted;
- Design: study design;
- Evaluation: the fixed battery used;
- Research type: type of study. Descriptions of "Evaluation" and "Research type" components followed Kapoor’s classification

For the batteries, the following information was extracted:

- target population;
- functions assessed;
- administration features (device used, format of instructions, responses, and scoring procedures, as well as tasks related to EF).

The analysis consisted of establishing the frequency of the extracted information. 

 Given that definitions of what constitutes EF vary among authors, it was anticipated that some tasks or tests from the batteries might be classifiable as EF components under a particular theoretical model, even if not explicitly labeled as such by the studies. Therefore, the tasks or tests within the identified fixed batteries were considered for this review if they aligned with the operational definitions of executive assessment paradigms proposed by Global Executive Function Initiative (GEFI)^7^, Diamond^1^, and Dias and Malloy-Diniz^8^. These operational definitions are detailed in Supplementary Material 1. 

 The selection of these three theoretical frameworks to categorize tasks as EF components was based on the following criteria:

- The Global Executive Function Initiative (GEFI) represents an international effort to standardize terminology and methodology in EF research, uniting researchers from diverse contexts and promoting widely recognized conceptual agreements^7^;
- Diamond’s model^5^ is the most frequently cited theoretical reference in contemporary EF studies and is widely adopted as a classificatory framework^5^;
- The model proposed by Dias and Malloy-Diniz^8^, presented in a reference work in Portuguese, provides theoretical and methodological contextualization for specific sociocultural realities, facilitating the inclusion of research paradigms applicable to the Brazilian population.

This adaptation is particularly important given the well-established intercultural variability of executive functioning, which demands instruments that are sensitive to the specific characteristics of different populations^18^. Thus, these three frameworks were considered complementary and representative of a conceptual gold standard for defining and classifying tasks as part of the executive function domain. 

### Procedure

 In accordance with PRISMA-ScR, the review was conducted in three main phases:

- Identification;
- Screening;
- Inclusion.

In Phase 1 (Identification), the database search was performed with the software Zotero™, which was used to organize and catalog the retrieved records. In Phase 2 (Screening), records that did not include the term "battery" in the full text were excluded using automated search tools within the software (Level 1 Screening). Additionally, documents that were not scientific articles — such as reviews, editorials, books, or conference proceedings — were excluded (Level 2 Screening). In Phase 3 (Inclusion), the full texts of the remaining studies were assessed for compatibility with the predefined eligibility criteria. The search, data extraction, and initial analysis were carried out by one of the authors. A second author reviewed all stages, including the inclusion criteria and the extracted data. Discrepancies between reviewers were discussed until consensus was reached. All extracted information from each study was recorded as notes within the corresponding Zotero™ files, facilitating traceability and systematization of the data during the analysis phases. 

## RESULTS

**Figure 1:**
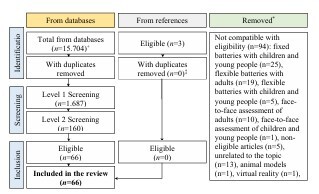
Preferred Reporting Items for Systematic reviews and Meta-Analyses (PRISMA) flowchart for identification, selection and inclusion of studies. Notes: *Removed as they are not compatible with eligibility at the Inclusion stage; +United States National Library of Medicine — PubMed (n=3,578), Scopus (n=4,409), Web of Science (n=4,578), Cochrane Library (Reviews) (n=53), PsychNet (n=3,079) e Scientific Electronic Library Online — SciELO (n=7); ‡Records were already present among the database records.

 The final sample consisted of 66 reviewed studies. Figure 1 presents the flowchart of the record selection process. Supplementary Material 2 describes the characteristics of the included studies^10,19-82^. Among the included studies, 42 fixed batteries were identified containing tasks or tests that could be classified under paradigms associated with executive functions (EF). Supplementary Material 3 presents the main characteristics of these instruments, based on the version described in the oldest study located for each battery^10,10,10,10,10,10,10,10,10,10,10,10,10,10,10,10,10,10,10,10,10^ The final sample consisted of 66 reviewed studies. Figure 1 presents the flowchart of the record selection process. Supplementary Material 2 describes the characteristics of the included studies^10,19-82^. Among the included studies, 42 fixed batteries were identified containing tasks or tests that could be classified under paradigms associated with executive functions (EF). Supplementary Material 3 presents the main characteristics of these instruments, based on the version described in the oldest study located for each battery^20,21,24,28,30,31,35,39,45,48,50,54,57,59,61,62,66,68,73-76,78-80,83-97^. The distribution of publications of the batteries by year reveals one battery published in 1998, 2001, 2003; two in 2005; one in 2006, 2012 and 2013; three in 2014; four in 2015; two in 2016, 2017 and 2019; three in 2020; two in 2021; four in 2022; three in 2023; and nine in 2024. Of the 42 batteries identified, only one was originally developed for use with both children/adolescents and adults (1/42; 2.4%). The remaining batteries were designed exclusively for adults and elderly (aged 18 years or older; 41/42; 97.6%). Furthermore, it was noted that four of these batteries are not recommended for use with older adults (individuals under the age of 60; 4/42; 9.5%). 

 None of the batteries consisted exclusively of tasks or tests classifiable as EF according to the method used in this review; all also included tasks not classifiable under the selected paradigms. Regardless of classification, none of the batteries claimed — based on their own terminology and definitions — to assess EF exclusively, but rather assessed EF alongside other cognitive abilities. Most batteries were originally applied with participants recruited in the United States (15 out of 42 batteries; 35.7%). Following in frequency were batteries recruiting from the United Kingdom (three batteries); Canada (three); China (three); France (three; 7.1%); Austria (two); South Korea (two; 4.8%); the United States and South Africa; Israel and Canada; Germany; Australia; Spain; Greece; Japan; the Netherlands; and Sweden (one battery each; 2.4%). 

 Most batteries were applied using computers (18/42; 42.9%), followed by those administered on tablets (9; 21.4%), touchscreen computers (four), smartphones (three), tablet or computer (three; 7.1%), tablet or touchscreen computer, tablet/smartphone/computer (two each; 4.8%), and one via tablet or smartphone (2.4%). The majority of batteries were initially designed for in-person application with professional supervision (24; 57.1%); 11 were self-administered (26.2%); five offered both in-person supervised and self-administered modes (11.9%); one provided either supervised synchronous remote administration or self-administration; and one provided supervised synchronous remote administration only (2.4%). Most batteries (17) presented on-screen written instructions without voice guidance (40.5%); other less common modes included on-screen instructions narrated simultaneously by the software (10; 23.8%), software-only narration (six; 14.3%), oral explanation by the professional during in-person administration (five; 11.9%), example videos (one; 2.4%), and mixed presentation (e.g., written for nonverbal tasks and narrated for verbal tasks) (two; 4.8%). One battery did not report how instructions were provided (2.4%). 

 Thirty-two batteries required direct motor responses on the device (76.1%); seven required verbal responses for verbal tasks and manual responses for nonverbal tasks (16.7%); one required manual responses and eye-tracking in one of its tasks; another involved responses captured by camera; and one did not report the response mode in its study (2.4%). Finally, 35 batteries generated results using automated scoring (83.3%); three used manual scoring (7.1%); two applied manual scoring for verbal tasks and automated scoring for nonverbal ones; and two did not report their scoring procedures (4.8%). None of the psychometric studies located described which EF model guided the selection of tasks for assessing EF. 

 Across the instruments, 176 tasks or tests were identified as being designed to assess executive functions (as categorized per the method of this review). Of these, 32 targeted inhibitory control, with the most common paradigm being Go/No-Go (16/32; 50%), followed by Stroop (14/32; 43.8%), Delay of Gratification (2/32; 6.3%), and Antisaccade (1/32; 3.1%). Regarding tasks with simultaneous emphasis on inhibitory control and cognitive flexibility, only one was found, developed under the Simon Effect paradigm. For cognitive flexibility, 54 tasks were identified, most of which followed the task-switching paradigm (29/54; 53.7%), followed by verbal fluency (14/54; 25.9%), Flanker (5/54; 9.3%), Dimensional Change Card Sort (DCCS) (4/54; 7.4%), and Design Fluency (2/54; 3.7%). For working memory, 31 tasks were found, of which 13 were based on the Digit Span paradigm, and 13 on Corsi Blocks (41.9%). Under the Continuous Performance Test (CPT) paradigm, 24 tasks were found that emphasized both working memory and inhibitory control. Thirteen tasks assessed working memory and cognitive flexibility simultaneously, under the Digit Symbol Substitution Test (DSST) paradigm. A total of 15 tasks were identified as targeting higher-level executive functions, most commonly based on the SAS paradigm (9/15; 60%), followed by Progressive Matrices (5/15; 33.3%) and Problem Solving (1/15; 6.7%). Supplementary Material 4 presents the resulting tasks. 

## DISCUSSION

 The present study aimed to identify internationally published fixed computerized batteries designed to assess EF in adults. The results revealed the absence of batteries built exclusively for this purpose, according to the criteria established by frameworks considered gold standards for the conceptualization and evaluation of EF. However, instruments were identified that, although broad in cognitive domains, include tasks that are classifiable as representative of EF based on the adopted guidelines. 

 Scoping reviews such as this in the present study may play a key role in organizing the available literature, enabling the mapping of existing instruments, the identification of gaps, and the expansion of access to assessment resources by professionals and researchers^15^. Furthermore, such reviews facilitate the selection of instruments appropriate for specific clinical contexts or age groups, contributing to more informed decisions about the suitability of measures for different populations^98^. 

 In this regard, the findings of this study offer an up-to-date synthesis of computerized batteries that include executive tasks, as used in the literature over the past ten years. These instruments have been applied in clinical and non-clinical samples, including individuals with subjective cognitive complaints, mild cognitive impairment (MCI), dementia, mood disorders (such as bipolar and depressive disorders), cognitive changes associated with long COVID, and cancer. 

 Thus, this mapping can serve as a reference tool for researchers and professionals working in computerized neuropsychological assessment, providing support for the critical selection of instruments based on their evaluative scope, application context, and potential psychometric limitations. However, the qualified use of these instruments requires additional studies on validation, cross-cultural adaptation, and diagnostic accuracy, particularly in diverse populations and non-hegemonic contexts. 

 Still, many tests in this format require technological familiarity, which may be difficult for this demographic^31^. Knowing that batteries have been developed with tasks that allow use with the elderly may help preserve cost-effectiveness while reducing the risk of applying instruments that do not adequately address health characteristics in this developmental stage. 

 It is worth noting that most batteries identified in this review are applicable to elderly people. The computerized application of neuropsychological instruments may represent a highly cost-effective alternative for assessing populations with limited access to specialized centers, such as individuals living in rural areas or those with physical limitations that hinder travel^99^. These conditions are particularly prevalent among older adults and have already motivated the development of remote cognitive assessment strategies^100^. However, the effectiveness of computerized assessment in this age group may be compromised by limitations related to technology use, as some elders demonstrate low familiarity with digital devices^31^. In this context, prior knowledge of computerized batteries composed of tasks that are feasible for older adults becomes strategic, allowing the maintenance of the logistical and economic advantages of this format without compromising sensitivity to the cognitive and functional particularities of this life stage. Therefore, the selection of appropriate instruments is crucial to ensure the validity of results and equity in access to neuropsychological assessment. 

 To deal with the elderly’s low technological familiarity with digital devices, the literature highlights solutions such as simplified interfaces, gamified tutorials with training phases, and hybrid on-site/remote support^48,74^. These strategies reduce technology-related bias, improve adherence, and enhance validity^74^. Adopting these approaches could strengthen digital health policies by ensuring that technological innovations in EF assessment expand, rather than restrict, access to evidence-based diagnostic and preventive care. 

 The identification of 42 fixed computerized batteries for EF assessment suggests a significant advancement in the development of neuropsychological instruments in this format, aligning with the trend of digitizing assessment processes^101^. It has already been discussed how the COVID-19 pandemic (2020–2023), by requiring educational, clinical, and occupational tasks to be performed online, also contributed to the growing number of professionals working in Teleneuropsychology^102^. The sharp increase in the number of new computerized batteries found by this review in 2024 compared to previous years may reflect the publication of studies conducted during the pandemic period, as a response to the increased demand for digitization. Maintaining this trend may be encouraged in order to access other advantages of this format, such as increased cost-effectiveness for examinees (e.g., regarding transportation), for examiners (e.g., through automated scoring and elimination of personal interference), and for public policy (e.g., through the adoption of new technologies) — benefits already highlighted in the literature prior to the pandemic^10,99^. 

 The heterogeneity observed in administration formats (in-person, self-administered, or remote), as well as in the devices used (computers, tablets, smartphones), reflects the adaptability of these batteries to different contexts. This flexibility is advantageous but also demands caution in interpreting results, as factors such as the testing environment and device type can influence performance^103^. In this sense, the development of standardized protocols and the conduct of cross-cultural validation studies become essential to ensure the reliability and validity of these instruments across populations and conditions. On the other hand, the high frequency of supervised in-person applications may represent a limitation to the trend toward remote assessment noted by the same author, especially in the post-pandemic context and for populations with restricted access to assessment centers. 

 None of the batteries identified explicitly stated the theoretical model of EF on which the selection or application of their tasks was based. However, all tasks listed could be classified based on the paradigms proposed by Diamond^1^, a model also widely used in the guidelines of the GEFI7 and by Dias and Malloy-Diniz^8^. Given this predominance, it is plausible that some tasks present in the batteries may also be compatible with other established theoretical models in the literature, such as those proposed by Lezak^103^, Kerr and Zelazo^104^, or Miyake et al.^105^, even if they were not directly categorized within those frameworks. As an additional example, a recent model^106^ included processing speed among EF, as assessed by batteries such as the Cognitive and Emotional Battery and NeuroScreen through psychomotor tests — Motor Tapping^20^ and Finger Tapping^60^, respectively. Describing theoretical models may benefit the development of future batteries, as this aspect could help professionals and researchers identify the best resources for evaluating abilities that may not be classifiable under a given model. 

 The predominance of batteries developed and applied in the United States and European countries, such as the United Kingdom, France, and Germany, reflects the leadership of these contexts in neuropsychological research and in the development of technologies applied to mental and cognitive health. However, the scarcity of batteries adapted or validated for diverse sociocultural contexts, such as Brazil, remains a challenge, given the importance of culturally sensitive instruments to ensure the ecological validity of assessments^18^. The literature emphasizes that cultural, educational, and linguistic differences can significantly impact performance on executive tasks, making cross-cultural adaptation and local norming of instruments essential^11^. 

 Dias et al.^5^ and Obradović et al.^7^ emphasize the need for terminological and methodological standardization in EF research to facilitate comparisons across studies and the integration of findings into clinical practice. The heterogeneity of definitions and paradigms hinders the construction of consensus and may negatively affect the diagnostic precision and clinical utility of instruments. Initiatives such as GEFI aim precisely to promote greater uniformity in the definition and operationalization of EF, recommending that future research and test development explicitly state the theoretical frameworks adopted^7^. 

 The lack of explicit reference to theoretical models of EF in most psychometric studies of the identified batteries reveals a significant conceptual gap. This omission compromises the transparency of the foundations guiding the construction and interpretation of instruments. As pointed out by Dias et al.^5^ and Obradović et al.^7^, standardization of terminology and methodology is essential to enable comparability across studies and integration into clinical practice. The heterogeneity in definitions and theoretical paradigms hinders scientific consensus and may reduce diagnostic precision while limiting the clinical applicability of the instruments. Initiatives such as GEFI have emphasized the importance of conceptual standardization and recommend that future research and test development clearly articulate the theoretical models they are based on. Such a practice not only strengthens the theoretical validity of measures but also facilitates replication and the careful use of instruments in different contexts and populations^7^. 

 Another relevant finding of this review was that none of the identified batteries assessed executive functions exclusively, and it was common for them to include tasks targeting other cognitive domains. This finding aligns with what is discussed in literature about the difficulty of isolating executive processes in neuropsychological tasks due to the multifaceted and interdependent nature of EF^107^. Diamond^1^ also argues that tasks traditionally classified as executive often involve demands related to working memory, attention, or language, complicating the construction of “pure” EF assessment instruments. Even GEFI^7^, which defines paradigms such as the Simon Effect as simultaneously targeting inhibitory control and cognitive flexibility, recognizes that variables like perceptual organization, general intelligence, and verbal comprehension of instructions may influence performance on executive tasks. 

 Thus, the presence of mixed tasks may be interpreted as a reflection of the inherent complexity of the EF construct and the need for comprehensive assessments that consider the overlap between cognitive domains. This discussion, clarified by the finding, suggests an opportunity for future test batteries and clinical evaluations to account for the role of other cognitive, psychomotor, and physiological functions on the the assessed results, rather than focusing solely on the main target of psychometric application. 

 This latter point aligns with criticisms raised by some authors^107,108^, who review the often limited ecological validity of neuropsychological tests applied in contexts that differ significantly from the examinees’ daily lives, where the overlap of cognitive demands, tasks, distractions, and goals is rarely replicated in controlled clinical or laboratory settings. Assessment tools based on daily life activities, such as the Hotel Task^109^ and Cognitive Estimation^110^, have been developed in response to similar criticisms. There is modest evidence that the ecological validity of items in computerized batteries enhances other forms of validity and reliability measures^111^. 

 In this review, at least 12 distinct batteries used computerized versions of the classic Trail Making Test with letters and numbers (task-switching paradigm; see Supplementary Material 4. Other batteries included tasks with abstract stimuli, such as the Stroop Test or card-sorting tasks in the DSST paradigm, which are frequently criticized for their low ecological validity^108^. In contrast, instruments like CogEvo^95^ and Cogsuite^66^ incorporated concrete everyday stimuli such as animals and familiar objects. 

 This distinction between abstract and ecological stimuli has practical implications: low-ecological-validity tasks may fail to detect deficits expressed outside the laboratory, while more realistic measures capture the clinical impact of executive dysfunctions^108^. In Brazil, this issue is exacerbated by the scarcity of standardized tests and, more critically, by the lack of ecologically valid computerized tools, underscoring the need for culturally adapted instruments that integrate scientific rigor with clinical applicability^109^. 

 The ecological validity of these different types of stimuli still lacks systematic investigation. Future studies should comparatively explore the incremental validity of computerized batteries using abstract versus contextually meaningful stimuli, focusing on sensitivity to detecting executive functioning in adults. Considering the relevance of executive functions to mental health, functional autonomy, and healthcare cost reduction^1^, this critical approach is essential for improving instruments with broader clinical and societal applications. 

### Limitations and future directions

 This review did not include an analysis of the psychometric properties of the identified batteries, which is a recommended focus for future studies. The selection considered only the first published study for each instrument, with the aim of mapping its origin and basic characteristics. This choice is justified by the fact that psychometric evidence is always relative to specific contexts and populations^112^, and this review did not restrict the target population in order to broaden the scope of the mapping. Additionally, the categorization of executive tasks was carried out exclusively based on the models of Diamond^1^, Obradović et al.^7^, and Dias and Malloy-Diniz^8^. As such, other functions frequently associated with EF — such as categorization, emotional regulation, initiative, and time management — may not have been represented. Future investigations should explore to what extent other tasks present in the mapped batteries address these functions, contributing to more sensitive assessments tailored to the specific demands of the individuals being evaluated. Finally, although most of the included batteries are applicable to adults and older adults, this review did not cover computerized instruments aimed at children and adolescents. Given the importance of assessing EF during early developmental stages — particularly for screening neurodevelopmental disorders —, it is recommended that future reviews include this population. 

 In conclusion, this review identified the existence of fixed, internationally available computerized batteries that include tasks aimed at assessing EF in adults and older adults. The findings revealed that:

- there are instruments applicable in remote, self-administered, or supervised in-person formats, enhancing their feasibility across various contexts;
- the widespread presence of computerized batteries reflects a consistent trend toward the digitization of neuropsychological assessment;
- the absence of explicit theoretical frameworks in the batteries compromises the conceptual delineation of EF and may hinder the identification of relevant cognitive functions that are not directly classified as executive; and
- the applicability of these batteries to older populations highlights opportunities for inclusive assessment in more vulnerable groups with limited access to specialized services. In light of these findings, future research is recommended to deepen the psychometric analysis of the mapped batteries, focusing on their validity, diagnostic sensitivity, and cross-cultural suitability. Such investigations may contribute to the more rigorous and effective use of these instruments, promoting cognitive assessments that are more accessible, standardized, and responsive to the specific demands of diverse populations and clinical settings.

## Data Availability

The datasets generated and/or analyzed during the current study are available from the corresponding author upon reasonable request.
