# Supplementary material for: Executive functions in adults: scoping review of computerized neuropsychological batteries
Source: Dement Neuropsychol. 2026 Mar 6;20:e20250374. doi: 10.1590/1980-5764-DN-2025-0374 (PMC12965716; doi:10.1590/1980-5764-DN-2025-0374)
Supplement: Supplementary Material 1 [file 1980-5764-dn-20-e20250374-md1.docx]

**Supplementary Material**

**Table S1**. Executive assessment task/test paradigms.

| **Construct** | **Paradigm** | **Paradigm description** | **Common measures** |
| --- | --- | --- | --- |
| Inhibitory control (response inhibition) + cognitive flexibility^*,+^ | Simon Effect^*,+^ | Three-block procedure. In the first (congruent) block, the participant is instructed to respond (e.g., press a button) on the same side as the target stimulus, to construct a prepotent response. In the second (incongruent) block, the participant is instructed to press a button on the opposite side of a stimulus (different from the first), to inhibit their prepotent response. In the third (mixed) block, the participant alternates between trials that present both stimuli, alternating between the different task rules. Performance in the second and third blocks suggests EF, since proficiency in the first block is common. | Accuracy or time |
| Inhibitory control: response inhibition^*,+^ | Go/No-Go^*,+^ | It is instructed to press a button when target stimuli appear and not to press it under other stimuli. | Accuracy or time |
|  | Delay Gratification^*,+^ | The aim is for the person being evaluated to expect a more rewarding result. | Acertos |
| Inhibitory control: suppression of interference^*,+^ | Stroop^*,+^ | You are instructed to say or select something that is the opposite (incongruent) to what is perceived. You need to overcome automatic tendencies to respond based on your perceptions and respond according to the objectives established in the task. | Accuracy or time |
|  | Antisaccade Task^+^ | It is instructed to inhibit (anti-saccade movement) the tendency to look in the direction of the emergence of a stimulus (pro-saccade movement). | Accuracy or time |
| Cognitive flexibility^*,+^ | Flanker task^+^ | The subject is instructed to pay attention to the target stimulus and ignore the flankers (surrounding distractor stimuli). A specific response is required if the target is congruent to the flankers (e.g., same direction or shape), incongruent (e.g., opposite directions), or neutral (unrelated; e.g., an arrow between dashes). | Accuracy or time |
|  | Unusual Uses or Design Fluency^+^ | The student is instructed to assign (e.g., draw or say) the most unusual use to a presented stimulus. The number of responses is measured by time. | Accuracy in time |
|  | Verbal Fluency^+^ | The student is instructed to name a number of items belonging to a semantic category (e.g., animals) or a phonological category (e.g., items beginning with S). ​​The two tasks can be performed successively. The author emphasizes that flexible minds will have an easier time finding atypical items. | Accuracy in time |
|  | Task-Switching^+^ | Alternate substitution between one response pattern, spontaneous or taught, for another is requested. | Accuracy in/or time |
| Working memory^*,+^ | Digit Span or Word Span^*,+^ | You are instructed to repeat a sequence of digits, words or phrases in the same order and/or in the reverse order of what is presented verbally. | Accuracy |
|  | Corsi Blocks or Dot Matrix^*,+^ | In a spatial distribution of stimuli (blocks, objects or points), one is instructed to remember, in the same or reverse order, the sequence of those presented (non-verbal presentation). | Accuracy |
|  | Self-Ordered Pointing^+^ | In a spatial distribution of boxes, you are instructed to find an item hidden in one of them. The objective is to not touch a previously chosen box again. | Accuracy |
| Working memory + sustained attention + inhibitory control (interference suppression)^*,+^ | CPT^+^ | A response (e.g., button press) is required whenever a target stimulus appears. Interference suppression may be required if distractors are present; sustainment may be required if the task is long (e.g., with long intervals between presentations); and working memory may be required if instructions are maintained (e.g., only when one stimulus follows another). | Accuracy or time |
| Cognitive flexibility + working memory‡ | DSST^‡^ | A sequence of symbols is presented, each with a corresponding number, and you are asked to complete a random sequence of distribution of these symbols with the numbers to which they correspond. | Accuracy in/or time |
|  | Symbol Search^‡^ | You are instructed to respond if one or more target items are found among distractor stimuli. | Accuracy in time |
|  | MOT^‡^ | You are instructed to follow one or more target stimuli between distractors while everyone moves. | Accuracy |
| Eductive reasoning^+^ | Progressive Matrices^+^ | In a matrix of images that logically complement each other and with a missing part, you are instructed to choose, from another list of images, the one that completes the matrix. | Accuracy |
| Planning^+,‡^ | SAS^‡^ | With a minimum number of moves, instruct to move balls through pins, one at a time, in order to compose a specific arrangement of balls on the pins presented in each item (Tower of London) or, instruct to move discs through pins, one at a time, in order to compose a specific arrangement of balls on the pins presented in each item, with the rule that a larger disc cannot be placed on top of a smaller one (Tower of Hanoi). | Accuracy |
| Problem-Solving^+^ | Mathematical Problems^+^ | Solving narrative mathematical problems. | Accuracy |

Note. *Definition by the Global Executive Function Initiative (GEFI)^7^. ^+^Definition by Diamond^1^. ^‡^Definition by Dias and Malloy-Diniz^8^. Abbreviations: CPT: Continuous Performance Test; DSST: Digit Symbol Substitution Test; MOT: Multiple-Object Tracking; SAS: Supervisory Attention System; SST: Symbol The GEFI^7^ suggests two paradigms as separate, but brought together by Diamond^1^ as forms of task-switching. The first one is the Flexible Item Selection Task (FIST), in which stimuli that vary in dimension (color, size, or shape) and value (e.g., small, medium, or large; blue, red, or yellow) are presented, and the student is instructed to identify different groupings of corresponding stimuli, alternating possibilities of association between dimensions and values. The second, the Dimensional Change Card Sort (DCCS), has three blocks: in the first, the student is instructed to sort cards according to dimension (e.g., color or shape); in the second, classify the same cards by another dimension (e.g., if previously by color, now by shape); and, in the third, flexibly alternate between the two dimensions following an additional rule for when to classify by each dimension (e.g., number of drawings).

**Table S2.** Characteristics of included studies.

| **Study** | **Sample** | **Phenomenon** | **Research type / design*** | **Battery used** |
| --- | --- | --- | --- | --- |
| Benoit et al. (2015)^19^ | Healthy adults or adults with schizophrenia (14-35) (*n* = 412) | Cognition under schizophrenia | Observational/cross-sectional | CogState |
| Gordon et al. (2015)^20^ | Adults from five unspecified countries (18-65) (*n* = 1,008) | Psychometric properties of a general cognitive screening battery (development) | Observational/cross-sectional | CEB |
| Jacova et al. (2015)^21^ | Adults with dementia, MCI or healthy (*n* = 27) | Psychometric properties of a battery for cognitive screening in elderly individuals (validity based on internal structure) | Observational/cross-sectional | C-TOC |
| Moore et al. (2015)^10^ | American adolescents (8-21) (*n* = 9,138) | Psychometric properties of a general cognitive screening battery (validity based on internal structure) | Observational/cross-sectional | PennCNB |
| Meskal et al. (2015)^22^ | Adults with meningioma (36-74) (*n* = 68) | Cognition of people with meningioma | Observational/cross-sectional | CNS VS |
| Turner et al. (2015)^23^ | New Zealand adults (20-28) (*n* = 22) | Cognitive changes under acute gas inhalation | Observational/cross-sectional | CNS VS |
| Assmann et al. (2016)^24^ | French adults (mean 57 years, no standard deviation reported) (*n* = 189) | Psychometric properties of a battery for cognitive screening in elderly individuals (reliability between different versions) | Observational/cross-sectional | NutriCog |
| Galletly et al. (2016)^25^ | USA adults (20-79) undergoing electroconvulsive therapy for depression (*n* = 63) | Effects of electroconvulsive therapy on cognition in depression | Experimental / intervencional | IntegNeuro |
| Hartung et al. (2016)^26^ | Healthy or TBI-injured young Americans (8–25) (*n* = 161) | Cognition under TBI | Observational/cross-sectional | PennCNB |
| Kuzmickienė e Kaubrys (2016)^27^ | Elderly l with (76.90 ± 5.20) or without (76.8 ± 6.61) AD (*n* = 75) | Cognition under AD | Observational/cross-sectional | CANTAB |
| Piper et al. (2016)^28^ | USA adults (18-34) (*n* = 44) | Young adult cognition | Observational/cross-sectional | PEBL |
| Yi et al. (2016)^29^ | USA (12-40) and Israeli (12-36) adults with 22q11.2 deletion syndrome (*n* = 214) | Cognition of Americans and Israelis | Observational/cross-sectional | PennCNB |
| Zhang e Feinstein (2016)^30^ | Canadian adults with traumatic brain injury (14-62) (*n* = 255) | Psychometric properties of a battery for cognitive screening in traumatic brain injury (development) | Observational/cross-sectional | BCBZF |
| Brenkel et al. (2017)^31^ | Canadian elders (≥ 65) (*n* = 45) | Psychometric properties of a battery for cognition (validity based on external measures) | Observational/cross-sectional | CBS |
| Holdnack et al. (2017)^32^ | USA adults with (47.4 ± 17.4) or without (38.6 ± 17.4) traumatic brain injury (*n* = 1,001) | Cognition under TBI | Observational/cross-sectional | NIH Toolbox |
| Kuiper et al. (2017)^33^ | Dutch adults (18-87) (*n* = 494) | Psychometric properties of a battery for cognitive screening of elderly individuals (reliability between different versions) | Observational/cross-sectional | CogState |
| Moore et al. (2017)^34^ | Highly educated US adults (25-56) (*n* = 96) | Psychometric properties of a general cognitive screening battery (development and validation) | Observational/cross-sectional | Cognition e WinSCAT |
| Völter et al. (2017)^35^ | Older adults (50-90) divided into groups with and without hearing loss (*n* = 120) | Psychometric properties of a battery for cognitive screening of elderly individuals with and without hearing loss (validity based on external measures) | Observational/cross-sectional | B-ALA |
| Cotter et al. (2018)^36^ | British adults with MS (22-70) (*n* = 90) | Cognition under MS | Observational/cross-sectional | CANTAB |
| de Meijer et al. (2018)^37^ | Australian adults with multiple sclerosis (47.5 ± 12.4) or healthy (35.2 ± 14.7) (*n* = 106) | Cognition under MS | Observational/cross-sectional | CogState (Brief Battery) |
| Gonçalves et al. (2018)^38^ | Portuguese adults (65-96) with AD (*n* = 137) | Psychometric properties of a battery for cognitive screening of elderly individuals with AD (validity based on external measures) | Observational/cross-sectional | CANTAB |
| Biagianti et al. (2019)^39^ | Healthy adults or adults with schizophrenia (16-65) (*n* = 391) | Psychometric properties of a battery for cognitive screening in schizophrenia (validity based on external measures) | Observational/cross-sectional | ONAs |
| Cerhan et al. (2019)^40^ | USA adults (30-76) with high-grade glioma (*n* = 40) | Psychometric properties of a battery for cognition in glioma (internal reliability) | Observational/cross-sectional | CogState |
| Golan et al. (2019)^41^ | US adults (25-55) with MS (*n* = 96) | Psychometric properties of a battery for cognitive screening in MS (validity based on external measures) | Observational/cross-sectional | Neurotrax |
| Helmstaedter et al. (2019)^42^ | German adults (35.7 ± 12.0) taking antiepileptic drugs (*n* = 55) | Psychometric properties of a battery for cognitive screening in epilepsy (validity based on external measures) | Observational/cross-sectional | NeuroCog FX |
| Lunter et al. (2019)^43^ | British adults with (18-77) or without (19-69) traumatic brain injury (*n* = 76) | Cognition under TBI | Observational/cross-sectional | CANTAB |
| Sicard et al. (2019)^44^ | Adults with (21.30 ± 0.18) or without (21.55 ± 0.25) concussion (*n* = 120) | Psychometric properties of a battery for cognitive screening in concussion (validity based on external measures) | Observational/cross-sectional | CogState |
| Chin et al. (2020)^45^ | Korean elderly (50-90) (*n* = 480) | Psychometric properties of a battery for cognitive screening in elderly individuals (development) | Observational/cross-sectional | Inbrain CST |
| Feinkohl et al. (2020)^46^ | German and Dutch elderly (65-89) (*n* = 45) | Psychometric properties of a battery for cognitive screening of elderly individuals (internal reliability) | Observational/cross-sectional | CANTAB |
| Nordenswan et al. (2020)^47^ | Finnish adults (21-46) (*n* = 233) | Psychometric properties of a battery for general cognitive screening (validity based on internal structure) | Observational/cross-sectional | CogState |
| Alegret et al. (2021)^48^ | Spanish adults (46-93) (*n* = 8,328) | Psychometric properties of a battery for general cognitive screening (development) | Observational/cross-sectional | NBACEtn |
| Barlow-Krelina et al. (2021)^49^ | Adults (8-29) with or without pediatric-onset MS (*n* = 141) | Psychometric properties of a battery for cognition in depression | Observational/cross-sectional | PennCNB |
| Bogert et al. (2021)^50^ | USA adults (18-60 years) with depression (*n* = 244) | Psychometric properties of a battery for cognition in depression (validity based on external measures) | Observational/cross-sectional | ReVeRe.D |
| Cui et al. (2021)^51^ | Elderly (72.5 ± 1.2) with or without normal tension glaucoma (*n* = 100) | Cognition under glaucoma | Observational/cross-sectional | NIH EXAMINER |
| MacAulay et al. (2021)^52^ | Adults of different socioeconomic statuses (57-87) (*n* = 121) | Psychometric properties of a general cognitive screening battery applied to elderly individuals (validity based on internal structure) | Observational/cross-sectional | NIH Toolbox |
| Scott et al. (2021)^53^ | South African Xhosa individuals with schizophrenia or healthy individuals aged approximately 38 years (*n* = 1,269) | Psychometric properties of a battery for cognition in the Xhosa population (validity based on external measures) | Observational/cross-sectional | PennCNB |
| Adolphe et al. (2022)^54^ | French adults (21-71) (*n* = 50) | Psychometric properties of a battery for cognitive screening in elderly individuals (development) | Observational/cross-sectional | OSCTB |
| Karlsen et al. (2022)^55^ | Norwegian adults (32.21 ± 13.10) (*n* = 75) | Psychometric properties of a battery for general cognitive screening (validity based on external measures) | Observational/cross-sectional | CANTAB |
| Kairys et al. (2022)^56^ | Elderly Americans with MCI (56-90) (*n* = 104) | Cognition of elderly people in a black community | Observational/cross-sectional | NIH Toolbox e CogState |
| Leemans et al. (2022)^57^ | Adults with brain metastasis (36-83) (*n* = 30) | Cognition under brain metastases | Observational/cross-sectional | Brainlab AG |
| Li et al. (2024)^58^ | Four groups of Taiwanese elderly (60+) with PD without cognitive deficits, with PD and CLL, with dementia, or healthy (*n* = 107) | Association of neuroimaging and plasma biomarkers with cognitive domain declines in patients with PD | Observational/cross-sectional | CANTAB |
| Moore et al. (2022)^59^ | Adults with or without bipolar disorder (18-65) (*n* = 66) | Psychometric properties of a battery for cognitive screening of people with bipolar disorder (validity based on external measures) | Observational/cross-sectional | NeuroUX |
| Robbins et al. (2022)^60^ | Thai individuals (10-23) with HIV (*n* = 100) | Psychometric properties of a battery for cognition in people with HIV (validity based on internal structure) | Observational/cross-sectional | NeuroScreen |
| van der Hurk et al. (2022)^61^ | Swedish adults (17-93) (*n* = 720) | Psychometric properties of a battery for general cognitive screening (norms) | Observational/cross-sectional | Mindmore – Remote Version |
| Glenn et al. (2023)^62^ | Japanese elderly with (77.5 ± 6.6) or without (72.7 ± 7.5) AD (*n* = 75) | Psychometric properties of a battery for cognitive screening of elderly individuals with AD (validity based on external measures) | Observational/cross-sectional | Neurotrack |
| Gu et al. (2023)^63^ | Three groups of Chinese elderly (60+) with dementia, MCI or healthy (*n* = 871) | Cognition under dementia | Observational/cross-sectional | CNCB |
| Ketvel et al. (2023)^64^ | Finnish adults (34-49) (*n* = 1,273) | Cognition under fatigue | Observational/cross-sectional | CANTAB |
| Revert-Alcántara et al. (2023)^65^ | Spanish adults (18-83) (*n* = 232) | Psychometric properties of a battery for general cognitive screening (validity based on external measures) | Observational/cross-sectional | Cognito |
| Root et al. (2023)^66^ | USA adult (39-65) cancer survivors (n = 357) | Psychometric properties of a cognitive screening battery for cancer survivors (validity based on external measures) | Observational/cross-sectional | Cogsuite |
| Sawada et al. (2023)^67^ | Japanese elderly (57-97) (*n* = 123) | Psychometric properties of a battery for cognitive screening of elderly individuals (validity based on external measures) | Observational/cross-sectional | CogEvo |
| Seok et al. (2024)^68^ | South Korean elderly with (77.9 ± 6.4) or without (74.1 ± 4.3) dementia (*n* = 185) | Psychometric properties of a battery for cognitive screening of dementia (validity based on external measures) | Observational/cross-sectional | Hellocog |
| Taylor et al. (2023)^69^ | USA adults with neurocognitive disorders (20–84) (*n* = 214) | Psychometric properties of a battery for cognition in people with dementia (validity based on external measures) | Observational/cross-sectional | ALLFTD-mAPP |
| An et al. (2024)^70^ | South Korean adults with subjective cognitive decline (69.69 ± 7.21), MCI (70.18 ± 7.80), or dementia (71.75 ± 9.09) (*n* = 254) | Psychometric properties of a battery for cognitive screening in elderly individuals (validity based on external measures) | Observational/cross-sectional | Inbrain CST^+^ |
| Gaynor et al. (2024)^71^ | USA adult (40-56) cancer survivors (*n* = 174) | Psychometric properties of a cognitive screening battery for cancer survivors (validity based on external measures) | Observational/cross-sectional | Cogsuite |
| Hoffmeister et al. (2024)^72^ | USA adults (*n* = 47) | Psychometric properties of a battery for cognitive screening in elderly individuals (validity based on external measures) | Observational/cross-sectional | Neurotrax |
| Li et al. (2024)^58^ | Australian seniors (51-84) (*n* = 404) | Association between finger-tapping task and general cognitive performance | Observational/cross-sectional | CANTAB |
| Maierhofer et al. (2024)^73^ | Austrian adults (19-90) (*n* = 70) | Psychometric properties of a general cognitive screening battery (validity based on external measures) | Observational/cross-sectional | INCP |
| Morrissey et al. (2024)^74^ | British elderly (70.19 ± 4.84) ​​(*n* = 32) | Psychometric properties of a battery for cognitive screening in elderly individuals (development) | Observational/cross-sectional | NeurOn |
| Pan et al. (2024)^75^ | Chinese adults (18–40) (*n* = 151) | Psychometric properties of a general cognitive screening battery (development) | Observational/cross-sectional | BOCAS |
| Patel et al. (2024)^76^ | Adults with cognitive impairment during long covid (*n* = 102) | Cognition under long covid | Observational/cross-sectional | LCCAB |
| Thienel et al. (2024)^77^ | Australian adults (46-71) (*n* = 141) | Association between brain morphology and cognitive performance | Observational/cross-sectional | Creyos [CBS] |
| Tsiaras et al. (2024)^78^ | Greek adults (18-70) (*n* = 169) | Psychometric properties of a battery for general cognitive screening (development) | Observational/cross-sectional | SAO |
| Woods et al. (2024)^79^ | USA elders (50-89) (*n* = 415) | Psychometric properties of a battery for cognitive screening of elderly individuals (development) | Observational/cross-sectional | CCAB |
| Zhang et al. (2024)^80^ | Chinese elderly (51-86) (*n* = 86) | Psychometric properties of a battery for general cognitive screening (development) | Observational/cross-sectional | CNCB |
| Bergman et al. (2025)^81^ | Swedish adults (20-79) (*n* = 149) | Psychometric properties of a general cognitive screening battery (internal reliability) | Observational/cross-sectional | Mindmore – Tablet Version |
| Wärn et al. (2025)^82^ | Swedish adults (18-89) (n = 52) | Psychometric properties of a general cognitive screening battery (validity based on external measures) | Observational/cross-sectional | Mindmore – Remote Version |

^*^According to Kapoor^17^. ^+^Later in this study called Seoul Cognitive Status Test (SCST). Abbreviations: B-ALA: Battery developed at the Institute for Work, Learning and Aging (ALA) [not named by the authors, but referred to as such in this review when mentioned]; Brainlab AG; CANTAB: Cambridge Neuropsychological Test Automated Battery; CBS: Cambridge Brain Sciences Battery; CCL: Cognitive Complaint Level; CNCB: Chinese Neuropsychological Consensus Battery; CNS VS: Central Nervous System Vital Signs; C-TOC: Cognitive Testing on Computer; Creyos: Creyos Battery [formerly mCBS]; HIV: Human Immunodeficiency Virus; IntegNeuro: IntegNeuro Cognitive Test Battery; MS: multiple sclerosis; NBACEtn: Neuropsychological Battery of Fundació ACE adapted for Teleneuropsychology; OSCTB: Open-Source Cognitive Test Battery [not named by the authors, but referred to as such in this review when mentioned]; PennCNB: The Penn Computerized Neurocognitive Battery; USA: United States of America.

**Table S3.** Batteries applied by the included studies (*n* = 42)

| **Battery** | **Authors** | **Cognitive screening** | **Functions^*^** | **Target examinees^+^** | **Country of sample recruitment** | **Device** | **Format** | **Instructions** | **Answers** | **Results** |
| --- | --- | --- | --- | --- | --- | --- | --- | --- | --- | --- |
| CANTAB | Sahakian et al. (1988)^83^ | In ND | Executive functions, verbal and visuospatial episodic memory, visuospatial working memory and emotion recognition | Elderly with AD (72.3 ± 6.6), PD with (64.2 ± 6.8) or PD without medications (61.3 ± 6.3) (n = 12) | UK | Computer with touchscreen | Supervised or self-administered in-person | Narrated by software | Manually on the device | Calculated by the software |
| PennCNB | Gur et al. (2001)^84^ | In general | Attention, verbal episodic memory, executive functions, fine motor skills, sensory perception and reasoning | Adults (18-54) (*n* = 92) | USA | Computer | Supervised in-person | Verbalized by the examiner | Manually on the device | Calculated by the software |
| Neurotrax | Dwolatzky et al. (2003)^85^ | In general | Attention, executive function, motor skills, memory (verbal and nonverbal), naming, information processing, and visuospatial skills | Healthy elderly (73.4 ± 8.0), with MCI (77.2 ± 6.4) and mild AD disease (80.6 ± 4.9) (*n* = 38) | Israel and Canada | Computer | Supervised in-person | Read on screen | Manually on the device | Calculated by the software |
| IntegNeuro | Paul et al. (2005)^86^ | In general | Executive functions, attention, verbal fluency, estimated intelligence, memory and sensorimotor functions | Adults and Australians (22-80) (*n* = 50) | USA | Computer with touchscreen | Supervised in-person | Read on screen and narrated by software | Manually on the device in nonverbal tasks and verbalized in verbal tasks | Calculated by the software |
| WinSCAT | Kane et al. (2005)^87^ | To austronauts | Attention, executive functions, processing speed, basic math skills, and visual and recognition memory | Astronaut adults | USA | Computer | Supervised in-person | Read on screen | Manually on the device | Calculated by the software |
| CNS VS | Gualtieri et al. (2006)^88^ | In general | Attention, executive functions, episodic memory and fine motor skills | Individuals (7-90) (*n* = 1,969) | USA | Computer | Supervised or self-administered in-person | Read on screen | Manually on the device | Calculated by the software |
| NeuroCog FX | Flechl et al. (2012)^89^ | In glioblastoma | Attention, language (verbal fluency), figural memory, verbal memory and working memory | Adults (24-71) with glioblastoma (*n* = 17) | Austria | Computer | Supervised in-person | Narrated by software | Manually on the device | Calculated by the software |
| NIH Toolbox | Gershon et al. (2013)^90^ | In general | Negative affect, positive affect, social support, attention, visual attention, hearing, inhibitory control and cognitive flexibility, dexterity, endurance, balance, stress and coping, muscular strength, executive function, vestibular function, language, locomotion, episodic memory, visual episodic memory, working memory, olfaction, taste, speed processing, visual speed processing, social relationships, somatosensory processing, and vision | English and/or Spanish speaking adults (3-85) (*n* = 47) | USA | Computer | Supervised in-person | Narrated by software | Manually on the device | Calculated by the software |
| Cognito | Ritchie et al. (2014)^91^ | In general | Attention (divided and focused), visuospatial skills, language (phonology, morphology, syntax, semantics, verbal fluency), memory (primary, secondary verbal, secondary visual, implicit, working), visuospatial reasoning, and reaction time | French adults (51.9 ± 17.3) (*n* = 135) | France | *Tablet or computer with touchscreen* | Supervised in-person | Narrated by software | Manually on the device | Calculated by the software |
| NeuroScreen | Robbins et al. (2022)^60^ | In HIV | Executive functions, verbal episodic memory, fine motor skills and processing speed | Adults (53.4 ± 7.0) HIV+ (*n* = 50) | USA and South Africa | *Smartphone* | Supervised in-person | Read on screen and narrated by software | Manually on the device | Calculated by the software |
| NIH EXAMINER | Kramer et al. (2014)^92^ | In general | Social cognition, cognitive flexibility (set shifting), verbal fluency, inhibition, insight, working memory and planning | Children and adolescents (3-17) and adults and seniors (18-94) (*n* = 1,248) | USA | Computer | Supervised or self-administered in-person | Read on screen | Manually on the device in nonverbal tasks and verbalized in verbal tasks | Calculated manually in verbal tasks or Calculated by the software in non-verbal tasks |
| CEB | Gordon et al. (2015)^20^ | In general | Attention, processing speed, motor coordination, executive functions and emotion identification | Adults from five unspecified countries (18-65) (*n* = 1,008) | NR | Computer | Self-administered | NR | NR | Calculated by the software |
| Cognition | Basner et al. (2015)^93^ | In general | Attention, executive functions, memory, spatial orientation, mathematical processing, emotion recognition, abstract reasoning, visuospatial reasoning, behavioral risk, psychomotor speed and sensorimotor speed | Adults (34-53) astronauts, astronaut candidates, or spaceflight controllers (*n* = 96) | USA | Tablet or Computer | Self-administered | Read on screen | Manually on the device | Calculated by the software |
| C-TOC | Jacova et al. (2015)^21^ | In general | Executive functions, memory, language, processing speed and visuoconstruction | Healthy elderly (71.6 ± 7.1; n = 13), with MCI (73.9 ± 8.7; n = 9) or with dementia (70.6 ± 7.4; *n* = 5) | Canada | Computer | Self-administered | Read on screen (tarefas não verbais) ou Narrated by software (verbais) | Manually on the device | Calculated manually |
| PEBL | Piper et al. (2015)^28^ | In general | Attention, memory and executive functions | Adults (18-22) (*n* = 189 in study I and *n* = 79 in study II) | USA | Computer | Supervised in-person | Read on screen | Manually on the device | Calculated by the software |
| BCBZF | Zhang e Feinstein (2016)^30^ | In TBI | Executive functions and information processing speed | Adults with TBI (14-62) (*n* = 255) | Canada | Computer | Supervised in-person | Verbalized by the examiner | Manually on the device | Calculated by the software |
| NutriCog | Assmann et al. (2016)^24^ | In general | Selective attention, mental flexibility, inhibition, associative, episodic, working and procedural memory, visual, planning and psychomotor speed | Adults (mean 57 years, no standard deviation described) (*n* = 189) | France | Computer | Supervised or self-administered in-person | Read on screen | Manually on the device | Calculated by the software |
| B-ALA | Völter et al. (2017)^35^ | To elders | Attention, executive functions, inhibition, short-term memory, long-term memory and working memory | Elderly (50-90) with moderate to severe hearing loss (*n* = 120) | Alemanha | Computer with touchscreen | Supervised in-person | Read on screen | Manually on the device | NR |
| CBS | Brenkel et al. (2017)^31^ | To elders | Attention, short-term memory, planning and reasoning | Elderly (mean 78 years, no standard deviation described) (*n* = 45) | Canada | Computer | Supervised in-person | Read on screen and narrated by software | Manually on the device | Calculated by the software |
| CNCB | Wang et al. (2019)^94^ | In ND | Attention, social cognition, executive functions, verbal episodic memory, language and perception | Adults and older adults with dementia, MCI or healthy (*n* from multiple studies) | China | Tablet or computer with touchscreen | Supervised in-person | Verbalized by the examiner | Manually on the device | Calculated by the software |
| ONAs | Biagianti et al. (2019)^39^ | In schizophrenia | Attention, executive function, learning, perception and socio-affective processing | Healthy adults or adults with schizophrenia (16-65) (*n* = 391) | USA | Computer | Self-administered | Read on screen | Manually on the device | Calculated by the software |
| CogEvo | Ichii et al. (2020)^95^ | In general | Attention, executive functions, memory, orientation and spatial cognition | Elderly (40-97) (*n* = 272) | Japan | Computer with touchscreen | Self-administered | Read on screen | Manually on the device | Calculated by the software |
| DCTB | Vermeent et al. (2020)^96^ | In general | Attention, executive function, working memory, processing speed, visuospatial processing, verbal fluency, learning and episodic memory | Adults (21-81) (*n* = 265) | Netherlands | Tablet | Supervised in-person | Read on screen (tarefas não verbais) ou Narrated by software (verbais) | Manually on the device in nonverbal tasks and verbalized in verbal tasks | Calculated manually in verbal tasks or Calculated by the software in non-verbal tasks |
| Inbrain CST | Chin et al. (2020)^45^ | In ND | Attention, executive functions, episodic memory, language and perception | Adults (50-90) with AD, MCI or subjective complaint, with approximately 12 years of education (76.2 ± 6.8) (*n* = 480) | South Korea | Tablet | Supervised in-person | Read on screen and narrated by software | Manually on the device | Calculated by the software |
| NBACEtn | Alegret et al. (2021)^48^ | In general | Attention, executive functions, memory, orientation (spatial, temporal and personal), language (verbal comprehension, naming, repetition), praxis, processing speed and visuospatial/visuoconstructive | Adults (46-93) (*n* = 8,328) | Spain | Tablet, smartphone or computer | Supervised, remote | Verbalized by the examiner | Shown to the camera in nonverbal tasks and verbalized in verbal tasks | Calculated manually |
| ReVeRe.D | Bogert et al. (2021)^50^ | In depression | Executive functions and episodic memory | Adults (18-49) college and older (*n* = 244) | USA | Tablet | Supervised in-person | Read on screen and narrated by software | Manually on the device in nonverbal tasks and verbalized in verbal tasks | Calculated by the software |
| Brainlab AG | Leemans et al. (2022)^57^ | In brain metastases | Attention and processing speed, memory and learning, verbal fluency, executive functions, fine motor skills | Adults with brain metastasis (36-83) (*n* = 30) | USA | Tablet | Supervised in-person | Read on screen and narrated by software | Manually on the device | Calculated manually |
| Mindmore | van der Hurk et al. (2022)^61^ | In general | Attention and processing speed, memory, language, visuospatial functions and executive functions | Adults (17-93) (*n* = 720) | Sweden | *Tablet or computer* | Supervised, remote (Remote Version) or Self-administered (Tablet Version) | Read on screen and Narrated by software | Manually on the device in nonverbal tasks and verbalized in verbal tasks | Calculated by the software |
| NeuroUX | Moore et al. (2022)^59^ | In BD | Attention, inhibitory control, executive function, episodic memory, recognition memory, working memory, processing speed | Adults with or without BD (18-65) (*n* = 66) | USA | Smartphone | Supervised in-person | Read on screen and narrated by software | Manually on the device | Calculated by the software |
| OSCT | Adolphe et al. (2022)^54^ | In general | Attention and working memory | Adults (21-71) (*n* = 50) | France | Computer | Self-administered | Read on screen | Manually on the device | Calculated by the software |
| ALLFTD-mAPP | Taylor et al. (2023)^69^ | In FTD | Executive functions, memory, language and motor skills | Adults with FTD (20-84) (*n* = 214) | USA | Smartphone | Self-administered | Read on screen | Manually on the device | Calculated by the software |
| Cogsuite | Root et al. (2023)^66^ | No câncer | Attention, executive functions, working memory, verbal ability, visuospatial ability, motor function and processing speed | Adult (39-65) cancer survivors (*n* = 357) | USA | Computer | Supervised or self-administered in-person | Read on screen | Manually on the device | Calculated by the software |
| Neurotrack | Glenn et al. (2023)^62^ | In ND | Visual recognition memory, processing speed, executive function and learning | Elderly individuals with (77.5 ± 6.6) or without (72.7 ± 7.5) AD (*n* = 75) | Japan | Tablet | Supervised in-person | Read on screen | Manually on the device and, in one task, eye tracking | Calculated by the software |
| BOCAS | Pan et al. (2024)^75^ | In general | Sustained attention, cognitive flexibility, sensorimotor skills, working memory, spatial navigation and processing speed | Adults (18-40) (*n* = 151) | China | Tablet | Supervised in-person | Read on screen | Manually on the device | Calculated by the software |
| CCAB | Woods et al. (2024)^79^ | In general | Attention, executive functions, visuospatial skills, language, memory and processing speed | Elderly (50-89) (*n* = 415) | USA | Tablet | Supervised in-person | Narrated by software | Manually on the device in nonverbal tasks and verbalized in verbal tasks | Calculated by the software |
| CNBC | Zhang et al. (2024)^80^ | In general | Attention, executive function, episodic memory and visuospatial skills | Elderly (51-86) (*n* = 86) | China | Computer | Self-administered | Read on screen and narrated by software | Manually on the device | Calculated by the software |
| CogState | CogState (2024)^§, 97^ | In general | Attention, social cognition, executive functions, working memory, visual and verbal memory, and processing speed | Initially, indigenous Australian populations (age NR) | Australia | Tablet, smartphone or computer | Self-administered | Narrated by software | Manually on the device | Calculated by the software |
| Hellocog | Seok et al. (2024)^68^ | In ND | A questionnaire part (Hellocog-Q) and a test part (Hellocog-T) for temporal orientation, memory (word learning, recall and recognition), executive function (trail making test), verbal fluency and naming | Elderly people with (77.9 ± 6.4) or without (74.1 ± 4.3) dementia (*n* = 185) | Coreia do Sul | Tablet | Supervised in-person | Read on screen | Manually on the device in nonverbal tasks and verbalized in verbal tasks | Calculated by the software |
| INCP | Maierhofer et al. (2024)^73^ | In general | Attention, executive functions, language, learning and memory, social cognition | Adults (19-90) (*n* = 70) | Austria | Tablet | Supervised in-person | Verbalized by the examiner | Manually on the device | NR |
| LCCAB | Patel et al. (2024)^76^ | In general | Attention, executive functions, language, learning and memory, social cognition | Adults with cognitive impairment during long covid (*n* = 102) | UK | Tablet ou smartphone | Supervised in-person | Read on screen and narrated by software | Manually on the device | Calculated by the software |
| NeurOn | Morrissey et al. (2024)^74^ | To elders | Attention, executive functions, episodic memory, visuospatial working memory, perceptual organization, spatial orientation and processing speed | Elderly (70.19 ± 4.84) ​​(*n* = 32) | UK | Tablet or computer | Self-administered | Read on screen e narradas em vídeo pelo *software* | Manually on the device | Calculated by the software |
| SAO | Tsiaras et al. (2024)^78^ | In general | Attention, executive functions, verbal memory, visual memory and working memory | Adults (18-70) (*n* = 169) | Greece | Computer | Audoadministrada | Read on screen | Manually on the device | Calculated by the software |

*According to categorization by the referenced studies. ^+^As per the referenced study, which is the oldest published source located for the instrument (cf. “Method”); other studies may exist with psychometric properties for other populations; age in parentheses (range or mean ± standard deviation, as reported by the authors). ^§^Developed in 1999^97^. Abbreviations (abbreviations are those used by the authors, except where reported, and when authors adopted titles already abbreviated by others, such as “TMT” for “Trail Making Test”): AD: Alzheimer's disease; AIM: Abstraction Inhibition and Working Memory; ALLFTD-mAPP: Advancing Research and Treatment for Frontotemporal Lobar Degeneration/Longitudinal Evaluation of Familial FrontoTemporal Dementia Subjects Mobile App; B-ALA: battery created at the Institute for Work, Learning and Aging (ALA) [not named by the authors, but referred to as such in this review when mentioned]; BCBZF: Brief Computerized Battery by Zhang and Feinstein^30^ [not named by the authors, but referred to as such in this review when mentioned]; BD: bipolar disorder; BMT: Block Maze Test; Brainlab AG: computerized battery developed by Brainlab AG [not named by the authors, but referred to as such in this review when mentioned]; BOCAS: Baguan Online Cognitive Assessment System; CANTAB: Cambridge Neuropsychological Test Automated Battery; CBS: Cambridge Brain Sciences Battery; CBTT: Corsi Block Tapping Test; CCAB: California Cognitive Assessment Battery; CCL: Cognitive Complaint Level; cDST: Computerized Digital [sic] Span Test; CEB: Cognitive and Emotional Battery [not named by the authors, but referred to as such in this review when mentioned]; CFT: Category Fluency Test; CGT: Cambridge Gambling Task; CNBC: Computerized Neurocognitive Battery for Chinese-Speaking Participants; CNCB: Chinese Neuropsychological Consensus Battery; CNSVS: Central Nervous System Vital Signs; Codesub: Code Substitution Test; Cogsuite: Enformia Cogsuite Battery; CPT [WinSCAT]: Running Memory Continuous Performance Test; CPT: Continuous Performance Test; CRPM: Computerized Raven’s Progressive Matrices; cSTT: Computerized Shape Trail Test; C-TOC: Cognitive Testing on Computer; Creyos: Creyos Battery; DCCS: Dimensional Change Card Sort; FTD: frontotemporal dementia; DICE: Dice 2-n Back; ND: neurodegenerative diseases; PD: Parkinson's disease; DSBT: Digit Span Backward Test; DSFT: Digit Span Forward Test; DSST: Digit Symbol Substitution Test; DST: Digit Symbol Test; FFT: Figure Fluency Test; FTD: frontotemporal dementia; FTT: Finger Tapping Test; IED: Intra-Extra Dimensional Set Shift; Inbrain CST: Inbrain Cognitive Screening Test; K-TMT-E: Korean-Trail Making Test–Elderly Version; LCCAB: Long COVID Assessment Battery; M2S: Delayed Matching to Sample; Math: Mathematical Processing; MTT: Multitasking Task; Neurotrack: Neurotrack Cognitive Assessment Battery (N-CAB); ND: neurodegenerative disease; NIH Toolbox: NIH [National Institutes of Health] Toolbox for Assessment of Neurological and Behavioral Function; NR: not reported; ONAs: Online Neurocognitive Assessments; OSCTB: Open-Source Cognitive Test Battery (not named by the authors, but referred to as such in the present study when mentioned); OSPAN: Operational Span Test; PAL: Paired Associates Learning; PASAT: Paced Auditory Serial Addition Test; PIT: Penn Inhibition Test; PVSAT-2: Paced Visual Serial Addition Test – 2-Second Trials; SAO: Self-Administered Online Battery; SART: Sustained Attention to Response Task; SAT: Shifting Attention Test; SDC: Symbol Digit Coding; SDMaT: Symbol Digit Matching Test; SDMT: Symbol Digit Modalities Test; SOC: Stockings of Cambridge; SS: Spatial Span; SST: Symbol Sorting Test; TBI: traumatic brain injury; TDT: Time Duration Test; TLT-s: Traffic Light Test–short; TMT: Trail Making Test (Forms A and/or B); UK: United Kingdom; USA: United States of America; VCT: Virtual Cooking Test; VFT: Verbal Fluency Test; VFA: Verbal Fluency – Animal Naming; VST: Visual Span Test; WinSCAT: Spaceflight Cognitive Assessment Tool for Windows.

**Tabela S4.** Table 4. Tasks or tests found in batteries classifiable as FE (*n* = 174)

| **Function** | **Paradigm** | **Tasks or tests categorized in paradigm** | ***n*** |
| --- | --- | --- | --- |
| Inhibitory control (response inhibition) + cognitive flexibility | Simon Effect | MTT/CANTAB | 1 |
| Inhibitory control: response inhibition | Go/No-Go | - Auditory Attention/COGNITO  - Balloon Collector (Stop Signal Task)/Cogsuite  - Feature Match/CBS  - Go/No-Go/CEB  - Go/No-Go/NeuroCog FX  - Go/No-Go/NeurOn  - Go-NoGo/Neurotrax  - Go/No-Go/OSCT  - Go/No-Go Task/LCCAB  - Go-No-Go/C-TOC  - Inverted Go/No-Go/NeuroCog FX  - M3/B-ALA  - PIT/PennCNB  - Test of Attentional Vigilance/PEBL  - TLT-s/INCP  - Visual Attention/COGNITO | 16 |
|  | Delay Gratification | - CGT/Cantab  - Decision Making Task/PennCNB | 2 |
| Inhibitory control: suppression of interference | Stroop | - Double Trouble/CBS  - Semantic Stroop/CCAB  - Stroop/ALLFTD-mAPP  - Stroop/CCAB  - Stroop/CNCB  - Stroop/CNSVS  - Stroop/DCTB  - Stroop Interference/Neurotrax  - Stroop Test/BCBZF  - Stroop Test/COGNITO  - Stroop Test/Mindmore  - Stroop Test/SAO  - Verbal Interference/CEB  - Verbal Interference/IntegNeuro | 14 |
|  | *Antisaccade* | Anti-Saccades/NIH EXAMINER | 1 |
| Cognitive flexibility | Flanker task | - Clownfish Commander (Attention Network Task)/Cogsuite  - Flanker/ALLFTD-mAPP  - Flanker/B-ALA  - Flanker Inhibitory Control and Attention Test/NIH Toolbox  - Flanker/NIH Toolbox | 5 |
|  | Unusual Uses or Design Fluency | - Design Fluency/CCAB  - FFT/INCP | 2 |
|  | Verbal Fluency | - Animal Fluency/Integneuro  - Category Fluency/NBACEtn  - Category Fluency/NIH EXAMINER  - CFT/DCTB  - Letter Fluency/IntegNeuro  - Letter Fluency/NBACEtn  - Phonemic Fluency/NIH EXAMINER  - Verbal Fluency/B-ALA  - Verbal Fluency/CCAB  - VFA/CNCB  - VFT for Animal Category/Hellocog  - Word Fluency/NeuroCog FX  - Words that Start with/Brainlab AG  - Zeppelin Race (Verbal Fluency Task)/Cogsuite | 14 |
|  | *Task-Switching* | - Auditory Task Switcher/ONAs  - Automatic Inhibition Subtest of the Syndrom-Kurztest/NBACEtn  - Click/NutriCog  - cSTT/CNBC  - DICE/INCP  - Executive Functioning/NeuroScreen  - Follow the Order/CogEvo  - IED/Cantab  - K-TMT-E/Inbrain CST  - Patch Points/Neurotrack  - Route 99/CogEvo  - SAT/CNS VS  - Similarities/C-TOC  - Switching of Attention Task/IntegNeuro  - Task-Switching/OSCT  - Task-Switching/OSCT  - TMT/BOCAS  - TMT/DCTB  - TMT/Hellocog  - TMT/Mindmore  - TMT-B/CNCB  - TMT-B/ReVeRe.D  - Trail Making Test/CogState  - Trail Making/C-TOC  - Trail Tasks/B-ALA  - Trail-Making Test/NeurOn  - Trail-Making Test/PEBL  - Trails A & B/CCAB  - Visual Task Switcher/ONAs | 29 |
|  | DCCS | - Barg Card Sort Test/PEBL  - Card Sort/ALLFTD-mAPP  - DCCS/NIH Toolbox  - Dimensional Set Shifting/NIH EXAMINER | 4 |
| Working memory | Digit Span or Word Span | - cDST/CNBC  - Digit Span/CBS  - Digit Span/CCAB  - Digit Span/CEB  - Digit Span/IntegNeuro  - Digit Span/NBACEtn  - Digit Span/NeuroCog FX  - Digit Span/PEBL  - DSBT/CNCB  - DSFT-DSBT/ReVeRe.D  - OSPAN/B-ALA  - Paired Associations/CBS  - Working Memory/NeuroScreen | 13 |
|  | Corsi Blocks or Dot Matrix | - CBTT/SÃO  - CopyKat/NeuroUX  - Flash Light/CogEvo  - LSWM Test/NIH Toolbox  - Memory Blocks/BOCAS  - Memory Matrix/NeuroUX  - Monkey Ladder/CBS  - SS/CANTAB  - VST/ Inbrain CST  - Span of Visual Memory/IntegNeuro  - Spatial Span/CBS  - Spatial Span/CCAB  - Spatial Span-Backwards/NeurOn, | 13 |
|  | Self-Ordered Pointing | - AIM/PennCNB  - BMT/ReVeRe.D  - M2S/WinSCAT  - PAL/CANTAB  - Token Search/CBS | 5 |
| Working memory + sustained attention + inhibitory control (interference suppression)^*,+^ | CPT | - Back/ALLFTD-mAPP  - Clock Judgement/BOCAS  - Continuous Addition/BOCAS  - Continuous Perfomance Task/IntegNeuro  - Continuous Performance Test/CEB  - CPT/CNS VS  - CPT/NIH EXAMINER  - CPT/WinSCAT  - Dot Counting/NIH EXAMINER  - Enumeration/OSCT  - Fractal 2-Back/Cognition  - Multiple-Objects Tracking Task/OSCT  - n-Back/NIH Examiner  - PASAT/Mindmore  - Psychomotor Vigilance Test/Cognition  - PVSAT-2/BCBZF  - Quick Tap 1 & 2/NeuroUX  - Quickbot Inspector (N-Back Task)/Cogsuite  - SART/SÃO  - Sustained Auditory Attention/ONAs  - Sustained Visual Attention/ONAs  - TDT/INCP  - Two Back Test/NeuroCog FX  - Working Memory Task/LCCAB | 24 |
| Cognitive flexibility + working memory | DSST | - DSST/CNCB  - DST/INCP  - Digit-Symbol Substitution Task/Cognition  - Oral Symbol Digit Test/NIH Toolbox  - SDC/CNS VS  - SDMaT/ReVeRe.D  - SDMT/BCBZF  - SDMT/Mindmore  - SST/ReVeRe.D  - Symbol-Digit Matching/C-TOC  - Symbol Match/Neurotrack  - Symbol-Number Coding/CCAB  - Zookeeper Match (Substituition Task)/Cogsuite | 13 |
|  | Symbol Search | - Processing Speed/NeuroScreen  - Pattern Comparison Processing Speed Test/NIH Toolbox  - Codesub/WinSCAT | 3 |
| Eductive Reasoning | Progressive Matrices | - CRPM/PennCNB  - Matrix Reasoning Test/Cognition  - Problem Solving/Neurotrax  - Visuospatial Reasoning/COGNITO  - Visual Reasoning Test/NIH Toolbox | 5 |
| Planning | SAS | - Hampshire Tree Task/CBS  - Maze/CEB  - Maze Task/IntegNeuro  - Maze A&B/NutriCog  - SOC/Cantab  - Square Puzzles/C-TOC  - Tower of Hanoi/Mindmore  - Tower-of-London/PEBL  - Unstructured Task/NIH Examiner | 9 |
| Problem-solving | Mathematical Problems | - Math/WinSCAT | 1 |

See Tables 1, 2 and 3 for acronyms and abbreviations.
